# Efficacy of dupilumab with concomitant topical calcineurin inhibitors treatment for preschool children with atopic dermatitis: a retrospective cohort study

**DOI:** 10.1080/07853890.2025.2449589

**Published:** 2025-01-06

**Authors:** Wu Liming, Kamran Ali

**Affiliations:** a Department of Dermatology, Affiliated Hangzhou First People’s Hospital, Westlake University School of Medicine, Hangzhou, China; b Department of Surgery, The Fourth Affiliated Hospital of School of Medicine, and International School of Medicine, International Institutes of Medicine, Zhejiang University, Yiwu, China

**Keywords:** Dupilumab, atopic dermatitis, pediatric AD, preschooler, topical calcineurin inhibitors, quality of life, preschool children

## Abstract

**Background/objective:**

Atopic dermatitis (AD) is a chronic, relapsing inflammatory skin disease that typically occurs in childhood/infancy and is associated with complications like extracutaneous atopic morbidity. Providing systemic treatment for pediatric AD patients with unmet comprehensive medical needs remains challenging. We present a cohort study describing the efficacy and safety of dupilumab combined with topical calcineurin inhibitors (TCI) in children with moderate-to-severe atopic dermatitis under the age of 6 years.

**Methods:**

A retrospective cohort study was conducted at a single center to analyze the use of dupilumab in combination with topical calcineurin inhibitors (TCI) in children aged 6 years and under moderate-to-severe AD that was inadequately controlled with topical therapy.

**Results:**

Overall, 23 preschool children (mean [*SD*] age, 4.78 [1.278] years); 10 boys (43.5%) and 13 girls (56.5%) received 300 mg dupilumab every four weeks and TCI. The primary outcome, the average Eczema Area and Severity Index (EASI) percentage reduction from baseline, was −70.85%. Significant improvement was also observed in secondary outcomes: caregiver-reported Peak Pruritus numerical rating scale (P-NRS) (−77.73%), Body Surface Area (BSA) (−62.11%), and Investigators Global Assessment (IGA) (−36.23%) at week 16. A 1–2 grade decrease in IGA after 16 weeks of treatment was achieved by 91.3% of patients. There was a significant improvement in P-NRS and EASI scores from baseline to week 16. Injection-site reaction (one patient) and facial redness (two patients) were recorded. No severe drug-related adverse events were observed.

**Conclusion:**

This study demonstrated that the combination of dupilumab and TCIs improved symptoms and quality of life in preschoolers with moderate-to-severe AD.

## Introduction

Atopic dermatitis (AD) is the most common chronic type 2 inflammatory skin disorder among infants and children [1], with a rising prevalence rate of AD of 15–30% in children and up to 2–10% in adults, and in pediatric population, 33% of children have moderate‐to‐severe AD [2]. AD significantly impacts the quality of life of both children and caregivers due to sleep disturbances, psychological distress, and stigmatizing skin lesions, and causing psychological distress in children [3]. The burden of AD is comparable to other non-dermatologic chronic childhood diseases, second only to cerebral palsy [4]. The impact on families can be greater than that of childhood type I diabetes. Itching and sleep disturbances are directly associated with the quality of life (QoL) of both the patients and their families [5].

Traditional topical medicines, such as topical calcineurin inhibitors (TCIs), topical corticosteroids (TCS), and PDE-4 inhibitors are commonly used for mild-to-moderate AD. For moderate-to-severe or refractory cases, systemic therapies are required. Dupilumab is the first fully human monoclonal antibody that blocks the IL-4/IL13-signaling (anti–interleukin-4 receptor α; IL-4R α) and inhibits receptor signaling downstream of the JAK-STAT pathway [6].

Systemic immunomodulating drugs, including cyclosporine, mycophenolate mofetil, azathioprine, and methotrexate, prompt off-label alternative therapeutic management options for moderate-to-severe pediatric AD. However, these drugs have limitations in efficacy and a low safety profile for patients with refractory disease, especially in preschoolers. Due to drug toxicity and severe side effects, these medications are not suitable for long-term use [7,8]. Treating AD is challenging, resulting in a high disease burden. Clinical trials analyzing the effectiveness and safety of dupilumab yielded significant outcomes. The U.S. Food and Drug Administration (FDA) recently approved dupilumab for treatment for the children aged 6 months to 5 years who have not responded to topical and systemic therapy or for whom topical therapy is not recommended.

In our single-center retrospective cohort, we observed significant improvement in clinical outcome measures, including EASI, IGA, and BSA). Additionally, caregiver-reported Peak Pruritus numerical rating scale (Pruritus-NRS). Dupilumab showed a promising safety profile superior to conventional immunosuppressive medications.

However, no published clinical data is available on the use of dupilumab in combination with TCIs in preschoolers. Here, we present a cohort study of preschoolers with moderate-to-severe AD treated with dupilumab and concomitant TCIs, examining its effectiveness as a novel treatment option for this age group. All the parents were informed of possible potential side effects, including injection site reactions, conjunctivitis, and an increased risk of infection. The written consent was provided by at least one of the guardians.

## Methods

### Study design and patients assessment

From March 2021 to May 2023, 23 preschoolers ages 2–6 with moderate-to-severe AD were enrolled in this study. The patients were treated with dupilumab and TCIs (tacrolimus 0.03% for facial lesions and 0.1% off-label for the upper and lower extremities and trunk). The institutional review board and ethics committee reviewed and approved the study protocol (IIT-20220301-0026-01). Data were retrospectively collected using a standardized data collection system. The main inclusion criteria were as follows: younger than six years of age with moderate-to-severe atopic dermatitis, no gender limitations, refractory to topical treatment (treated for at least 1 year), and diagnosed according to the Hanifin-Rajka criteria. All patients had chronic AD, which was uncontrolled with topical medications.

All parents were informed of the possible side effects, and written consent was obtained, stating that the information on clinical history could be used for research. All patients had eczema since birth and were recalcitrant to traditional treatment, affecting their quality of life and growth. They were treated with a 300 mg loading dose of dupilumab, followed by 300 mg every 4 weeks (q4w), and TCI twice a day until the lesion improved, and then switched to once a day to maintain the treatment. We collected data on patients’ age, sex, and personal/family history of atopic comorbidities (asthma, rhinitis, food, and environmental allergy). Clinical response was evaluated by Investigator’s Global Assessment (IGA), Body Surface Area (BSA), Eczema Area and Severity Index Score (EASI), and caregiver-reported Peak Pruritus numerical rating scale (P-NRS) at the time of dupilumab initiation and recent follow-up visit. Patients had been treated for at least 16 weeks with four injections when writing this report and were still on maintenance treatment with biweekly follow-up.

Treatment-emergent adverse events (TEAEs) and serious adverse events (SAEs) were also measured. Due to the increased incidence of conjunctivitis, injection-site reactions, and facial eczema, patients were warned of these potential side effects during dupilumab treatment. One of the parents or legal guardians signed the written informed consent. This study complied with the principles of the Declaration of Helsinki.

### Statistical analysis

Descriptive statistics were calculated based on the frequency and percentage change for categorical variables and the mean ± standard deviation (*SD*) for continuous variables. We analyzed the data using SPSS for all statistical analyses.

## Results

**Figure 1.**
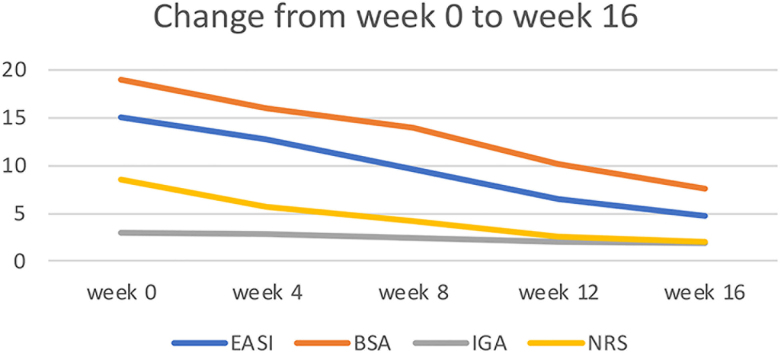
The change in baseline scores at week 16. Efficacy outcome of eczema severity index from baseline characteristics to week 4, 8, 12, and 16 in mean (*SD*): EASI, NRS, BSA, and IGA.

A total of 23 patients (mean [*SD*] age, 4.78 [1.278] years); 10 boys (43.5%) and 13 girls (56.5%) were evaluated in this study. The baseline characteristics expressed as mean (*SD*) were EASI 14.98 (7.57), P-NRS 8.47 (1.44) with a range of 3.00–10.00, BSA 18.95 (11.11), IGA 2.95 (0.70) with a range of 2.00–4.00. Nine patients (39.13%) reported with frequent comorbidities, such as rhinitis, asthma, urticaria, and food allergies. Eight patients (34.78%) had a family history of atopic comorbidities. The baseline patient characteristics are shown in Table 1. There was a statistically significant improvement in AD symptoms, leading to a better quality of life and sleep. The mean reduction in the AD parameters from baseline to week 16 was statistically significant. The change from baseline to week 16 expressed in mean (*SD*) and 95%CI were: EASI 4.79 (4.23), 95%CI; 1.07 (7.96–12.42), P-NRS 2.04 (2.01), 95%CI; 0.33 (5.74–7.12), BSA 7.54 (6.25), 95% CI; 1.35 (8.59–14.22), IGA 1.87 (0.69), 95% CI; 0.14 (0.80–1.37), respectively (Table 2) (Figure 1). The *p*-value was <0.05.

**Table 1.** Baseline demographics and disease characteristics.

Characteristics	Mean *SD*	range
Age, years, mean ± *SD*	4.78 (1.278)	(2–6)
Sex, *n* (%)		
Male	10 (43.5%)	
Female	13 (56.5%)	
Family history of rhinitis/asthma/urticaria/atopic dermatitis, *n* (%)	8 (34.78%)	
Patients’ history of allergic rhinitis/asthma/urticaria, *n* (%)	9 (39.13%)	
EASI (range 0–72), mean ± *SD*	14.98 (7.57)	6.00–33.00
P-NRS (range 0–10), mean ± *SD*	8.47 (1.44)	5.00–10.00
BSA (%), mean ± *SD*	18.95 (11.11)	7.00–51.00
IGA (range 0–4), mean ± *SD*	2.95 (0.70)	2.00–4.00

EASI: Eczema Area and Severity Index; P-NRS: caregiver-reported Peak Pruritus numerical rating scale; BSA; body surface area; IGA: Investigator’s Global Assessment; *n*: number of subjects; *SD*: standard deviation.

**Table 2.** Efficacy and percentage outcome at week 4, 8, 12, and 16.

Outcome			Mean (*SD*)	
EASI at baseline, mean ± *SD*			14.98 (7.57)	
EASI at week 4, mean ± *SD*			12.77 (7.83)	
EASI at week 8, mean ± *SD*			9.57 (6.80)	
EASI at week 12, mean ± *SD*			6.43 (5.55)	
EASI at week 16, mean ± *SD*			4.79 (4.23)	
EASI change from baseline to week 16, SE 95% CI			1.07 (7.96–12.42)	
P-NRS at baseline, mean ± *SD*			8.47 (1.44)	
P-NRS at week 4, mean ± *SD*			5.63 (2.30)	
P-NRS at week 8, mean ± *SD*			4.13 (2.31)	
P-NRS at week 12, mean ± *SD*			2.60 (2.31)	
P-NRS at week 16, mean ± *SD*			2.04 (2.01)	
P-NRS change from baseline to week 16, SE 95% CI			0.33 (5.74–7.12)	
BSA at baseline, mean ± *SD*			18.95 (11.11)	
BSA at week 4, mean ± *SD*			15.95 (13.04)	
BSA at week 8, mean ± *SD*			13.91 (9.94)	
BSA at week 12, mean ± *SD*			10.18 (9.56)	
BSA at week 16, mean ± *SD*			7.54 (6.25)	
BSA change from baseline to week 16, SE 95% CI			1.35 (8.59–14.22)	
IGA at baseline, mean ± *SD*			2.95 (0.70)	
IGA at week 4, mean ± *SD*			2.78 (0.67)	
IGA at week 8, mean ± *SD*			2.43 (0.78)	
IGA at week 12, mean ± *SD*			2.00 (0.67)	
IGA at week 16, mean ± *SD*			1.87 (0.69)	
IGA change from baseline to week 16, SE			0.14 (0.80–1.37)	
95% CI				
Outcome	Total number of subjects	Mean percentage (%)	*SD*	Minimum–maximum
EASI percentage change from baseline to week 16	23	−70.85%	16.42	−96.67 to −37,76
P-NRS percentage change from baseline to week 16	23	−77.73%	21.01	−100.00 to −30.00
BSA percentage change from baseline to week 16	23	−62.11%	18.03	−92.31 to −20.00
IGA percentage change from baseline to week 16	23	−36.23%	23.78	−66.67 to 50.00
IGA downregulation by ≥2 grades or greater	23	69.6%		
IGA downregulation by 1 grade	23	21.7%		

EASI: Eczema Area and Severity Index; P-NRS: caregiver-reported Peak Pruritus numerical rating scale; BSA; body surface area; IGA: Investigator’s Global Assessment; CI: confidence interval; *n*: number of subjects; *SD*: standard deviation; *SE*: standard error; Mean Percentage (%): mean percentage change from baseline to week 16.

The average EASI percentage improvement from baseline was −70.85%. Similarly, significant improvements were observed in caregiver-reported Peak Pruritus numerical rating scale (P-NRS), BSA, and IGA, with percentage improvements of −77.73, −62.11, and −36.23%, respectively at week 16. There was a significant decrease in P-NRS and EASI scores from baseline to week 16 (Table 2).

A decrease in IGA by 69.6% with ≥2 grades or greater decrease in IGA and 21.7% with a 1-grade decrease after 16 weeks of treatment was recorded (Table 2). However, one patient’s IGA score increased by one grade, whereas another patient did not show any improvement. The incidence of adverse effects was also evaluated. One patient experienced an injection site reaction that resolved without special treatment. Two patients complained of facial redness and burning sensation and continued treatment with a combination of TCI and moisturizer; the symptoms improved without special treatment. No allergic conjunctivitis was observed at baseline or during treatment. No drug-related severe adverse events (SAE) were observed. Overall, statistically significant improvement were observed in children treated with dupilumab.

## Discussion

Dupilumab, a biological drug targeting the type 2 inflammatory pathway, is the first FDA-approved biological treatment for AD in children and adults. It inhibits IL-4/IL-13, resulting in the downregulation of JAK-STAT pathway receptor signaling and type-2 immunity [9]. The benefits are multidimensional including improved skin symptoms, reduced symptom intensity and frequency, and enhanced health-related quality of life [10–13]. Anti-JAKs have emerged as potent drugs for treating atopic dermatitis in adolescents [14,15]. However, there is a significant unmet need for effective, long-term therapeutic options for children under 6 years of age. Our retrospective cohort study included 23 preschoolers with AD treated with dupilumab and TCIs for at least 16 weeks.

Currently, a multicenter phase II open-label study for pediatric patients under six years of age, such as Liberty AD PRESCHOOL (NCT03346434), is evaluating the safety, pharmacokinetics, and efficacy of dupilumab in patients aged six months to <6 years with severe AD [16]. A phase II, open-label study involving children aged six months to six years with uncontrolled severe atopic dermatitis investigated single-dose dupilumab in two groups based on sequential age and dosing. The older cohort included children aged ≥2 to <6 years treated with 3 mg/kg dupilumab, and the younger cohort included children aged ≥6 months to <2 years treated with 6 mg/kg dupilumab. Efficacy and safety assessments were conducted after a single subcutaneous injection of dupilumab. Three weeks after starting treatment with 3 and 6 mg/kg dupilumab, the mean EASI score decreased by 44.6 and 49.7%, respectively, in the older cohort and by 42.7 and 38.8% in the younger cohort. Peak pruritus scores decreased by 22.9 and 44.7%, respectively, in the older cohort and by 11.1 and 18.2%, respectively, in the younger cohort. At week 4, both age groups showed improvement in efficacy outcomes at the lower dose [16]. Our study expands upon the preschooler data by suggesting that average percentage of successful therapy decreased from baseline to EASI (−70.85%), P-NRS (−77.73%), BSA (−62.11%), and IGA (36.23%) at week 16.

There are insufficient clinical data regarding the safety and efficacy of dupilumab in the preschool population. In the LIBERTY AD CHRONOS trial, 39% of adults with moderate-to-severe atopic dermatitis achieved both IGA 0/1 and 2 points or greater improvement at week 16 with dupilumab plus topical corticosteroids, compared to 12% with placebo plus topical corticosteroids [12]. A clinical trial in Chinese patients with moderate-to-severe atopic dermatitis showed a 26.8% reduction in the IGA score of ≥2 points from baseline in the dupilumab group and 4.8% in the placebo group [17]. However, our cohort study showed successful treatment in preschoolers, with 69.6% having ≥2 grade or greater decrease in IGA and 21.7% having a 1 grade decrease in IGA after 16 weeks of dupilumab plus TCI. Recently, Da et al. and Ali et al. reported a case series of moderate-to-severe atopic dermatitis in children under six years of age treated with dupilumab. Patients showed a significant reduction in severity scores; however, one in three patients had an injection site reaction [18].

Furthermore, an open-label extension study carried out to evaluate the safety and efficacy of dupilumab in patients with AD aged between 6 months and 18 years (NCT02612454) [19]. Adults and pediatric patients with moderate-to-severe AD were enrolled in three prospective observational studies (NCT03549416, NCT03428646, and NCT03411837) to evaluate dupilumab the efficacy and safety of dupilumab for AD, as well as its comorbidities. The results of these studies are highly anticipated because Th2 inflammation is a determinant of AD in children even more so than in adults [20].

Safety concerns are significant when treating preschool children treated with new drugs, such as dupilumab. However, children and adults in all AD trials had similar safety results, with no severe treatment-related side effects observed [11,13,21]. According to previous studies, injection site reactions, conjunctivitis, headache, and local herpes simplex infection are the most commonly observed side effects [12,22,23]. It has been speculated that dupilumab use for treating allergic diseases, especially in children, may be limited by the higher frequency of conjunctivitis [11,23,24]. Dupilumab is generally well tolerated, with fewer toxicity concerns than other systemic AD medications, which is promising. Furthermore, common side effects are mild and easily manageable [25]. In our study, there were no severe adverse events or systemic hypersensitivity to dupilumab in children under six years of age, and the safety profiles of dupilumab among adults, adolescents, and children over six years of age were comparable [11,12,26–28]. We believe that data from extensive studies of children in the future will clarify this issue.

Our study supports that dupilumab combined with TCI is helpful and effective in children under six years. All patients experienced significant changes in signs and symptoms and subsequent improvement in dermatitis lesions within 16 weeks of treatment. However, there were some study limitations, including small sample size, age range, short follow-up period, and lack of a control group that used either only topical therapy or only dupilumab treatment. Additionally, laboratory tests, such as serology and skin colonization testing of *Staphylococcus aureus* was not performed before and after treatment, so relevant data are lacking.

## Conclusions

Dupilumab combined with topical calcineurin inhibitors showed clinically significant and meaningful results in preschoolers with moderate-to-severe AD. Significant improvement in AD symptoms, including pruritus, was achieved. The treatment was well tolerated, with no severe drug-related side effects, infections, or systemic hypersensitivities. This study yielded promising results that could directly affect future clinical practice after weighing the pros and cons. We speculate that dupilumab can overcome these challenges in preschool children with moderate-to-severe AD. Early intervention can potentially prevent atopy and chronic inflammation.

## Data Availability

The data supporting the findings of this study are available from the corresponding author upon reasonable request.
